# Function and regulation of Rab GTPases in cancers

**DOI:** 10.1007/s10565-024-09866-5

**Published:** 2024-05-02

**Authors:** Shouying Xu, Bin Cao, Ge Xuan, Shu Xu, Zihao An, Chongying Zhu, Lin Li, Chao Tang

**Affiliations:** 1 National Clinical Research Center for Child Health of the Children’s Hospital, Zhejiang University School of Medicine, Hangzhou, 310052 China; 2 Zhejiang Provincial Center for Disease Control and Prevention, Hangzhou, China; 3 Department of Gynaecology, Ningbo Women and Children’s Hospital, No.339 Liuting Road, Ningbo, 315012 China; 4 The Department of Obstetrics and Gynecology, Ruijin Hospital, Shanghai Jiaotong University School of Medicine, 197 Ruijin 2nd Road, Shanghai, 200025 China; 5 Department of Urology, Third Affiliated Hospital of the Second Military Medical University, Shanghai, 201805 China

**Keywords:** Rab GTPases, Vesicle transport, Tumor oncogene, Tumor suppressor

## Abstract

The Rab small GTPases are characterized by the distinct intracellular localization and modulate various endocytic, transcytic and exocytic transport pathways. Rab proteins function as scaffolds that connect signaling pathways and intracellular membrane trafficking processes through the recruitment of effectors, such as tethering factors, phosphatases, motors and kinases. In different cancers, Rabs play as either an onco-protein or a tumor suppressor role, highly dependending on the context. The molecular mechanistic research has revealed that Rab proteins are involved in cancer progression through influences on migration, invasion, metabolism, exosome secretion, autophagy, and drug resistance of cancer cells. Therefore, targeting Rab GTPases to recover the dysregulated vesicle transport systems may provide potential strategy to restrain cancer progression. In this review, we discuss the regulation of Rab protein level and activity in modulating pathways involved in tumor progression, and propose that Rab proteins may serve as a prognostic factor in different cancers.

**Graphical Abstract:**

• Rab GTPases together with the cognate effectors coordinates the dynamics of trafficking pathway and ensures the spatiotemporal regulation of vesicle trafficking.

• Functional impairments of the regulatory network of vesicle trafficking are associated with tumorigenesis.

• Rab proteins play oncogenic or tumor suppressor roles in different cancers depending on context.

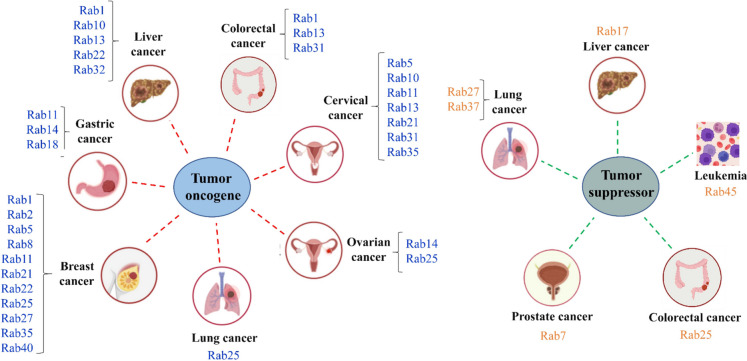

## Introduction

**Fig. 1:**
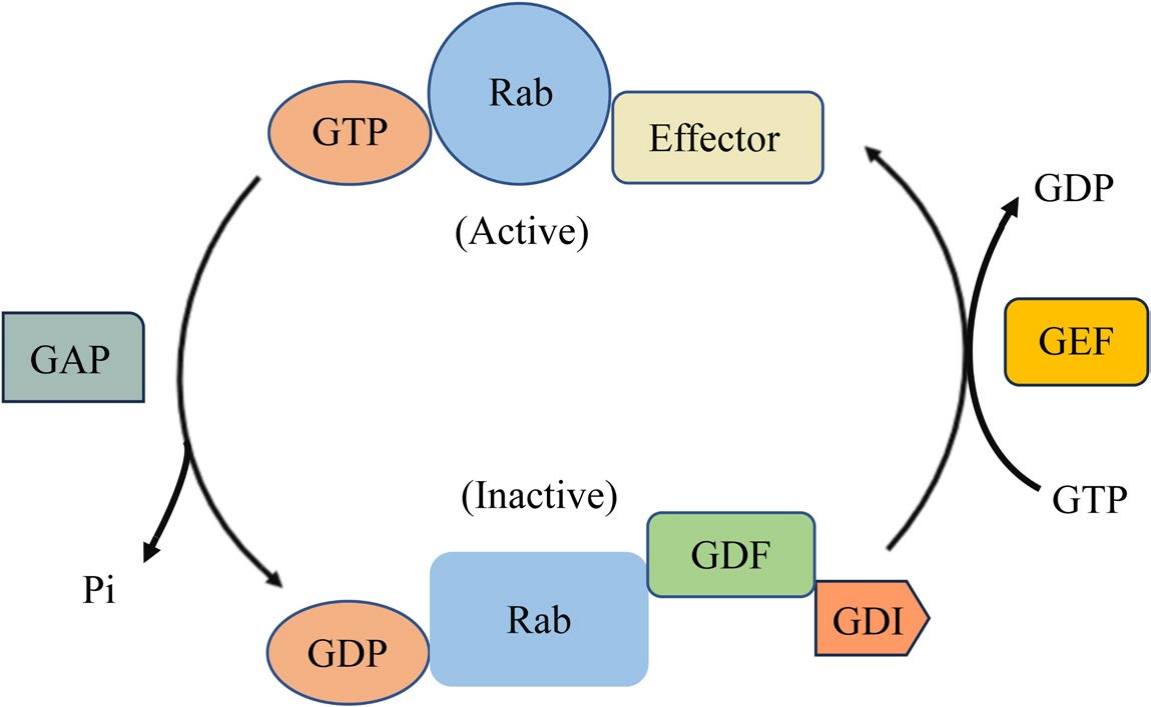
Schematic illustration of the Rab GTPase cycle

Rab GTPases, as the largest branch of the Ras superfamily of proteins, function as regulators of vesicle transport, protein trafficking, membrane targeting and fusion. In humans, there are more than 70 different Rab proteins that are localized to distinct intracellular membranes (Schwartz et al. 2007). Rab proteins are alternate between GTP bound state (active state) and GDP bound state (inactive state), which are modulated by guanine nucleotide exchange factors (GEFs) and GTPase-activating proteins (GAPs), acting as molecular switches to regulate vesicular trafficking from donor membrane budding toward acceptor membrane fusion along cytoskeleton as well as membrane fusion at the target compartment (Fig. 1) (Cernochova et al. 2016; Burk and Pasterkamp 2019).

Cooperation with their effectors, such as GEFs, GAPs and guanine dissociation inhibitors (GDIs) together with tethering factors and Soluble N-ethylmaleimide sensitive factor attachment protein receptors (SNAREs), Rab proteins are crucial for mediating vesicular traffick from donor membrane budding toward acceptor membrane fusion as well as specialized pathways regulating cell growth, survival and apoptosis (as summarized in Table 1) (Burk and Pasterkamp 2019; Seabra and Coudrier 2004; Markgraf et al. 2007; Ohya et al. 2009). Most of the Rab proteins that mediate secretory and endosomal membrane transport and autophagosome biogenesis are important ingredients of vesicle transport machinery (Tzeng and Wang 2016). In lysosomes and exocytosis, the changes in vesicle transport pathways that mediates cargo delivery to the cell surface, endocytosis, recycling and degradation, contribute to the changes in expression of Rab GTPase (Lamber et al. 2019). Thus, the cooperation between Rab proteins and cognate effectors in regulating vesicle transport pathways exhibits the influences of Rab proteins on tumor progression and malignancy (Tzeng and Wang 2016). Rab GTPases play important roles in the regulation of cancer cell biology, including proliferation, migration, invasion, autophagy, exosome secretion, metabolism and drug resistance (Li and Marlin 2015). These processes are regulated by multiple coordinated signaling pathways, among which the MAPK pathway and PI3K pathway play important roles in increasing cell survival and suppressing cell apoptosis (Gopal Krishnan et al. 2020).

**Table 1:** The correlation of specific Rabs with their regulator factors and effectors

Regulator factors/Effectors	Protein name	Reference(s)
GAP for Rab3	Rab3GAP1 and Rab3GAP2	Handley et al. 2013)
GEF for Rab5	RIN1	Stenmark 2009)
GEF for Rab32, -38	HSP1, HSP4	Gerondopoulos et al. 2012)
GDI	GDI1	D'Adamo et al. 1998)
GGT	Rab GGTa	Detter et al. 2000)
REP	REP1	Andres et al. 1993)
Effectors for Rab3A	Rabphilin	Tsuboi and Fukuda 2005)
Effectors for Rab5	Class I PI 3-kinase, Class III PI 3-kinase, EEA1, rabenosyn 5, rabankyrin 5	Stenmark 2009)
Effectors for Rab6	COH1/VPS13b, KIF20A, bicaudal D1	Zhen and Stenmark 2015)
Effectors for Rab7	RILP, Class III PI 3-kinase, WDR91	Jordens et al. 2001; Xing et al. 2021)
Effectors for Rab9	TIP47	Carroll et al. 2001)
Effectors for Rab8, -11	Myosin Vb	Knowles et al. 2014)
Effectors for Rab1, -3, -5, -6, -8, -13, -22	OCRL	Fukuda et al. 2008)
Effectors for Rab11, -25	Rab11FIP2, Rab11FIP5	Fukuda et al. 2008; Hales et al. 2002)
Effectors for Rab27a	Melanophilin, granuphilin, Myosin Va, Munc13-4, SlP2	Stenmark 2009)
Effectors for Rab34	RILP	Progida et al. 2006)
Effectors for Rab1, -3, -5, -6, -8, -9, -22, -35	INPP5B	Fukuda et al. 2008)

Here, this review mainly introduces the mechanisms regarding with Rab small GTPases in modulating progression of multiple cancers and discusses the relationship between Rab proteins and vesicle transport, highlighting that deep investigation of Rab proteins is of potential guiding significance for the early diagnosis and treatment of certain human cancers.

## Rab small GTPase family

**Fig. 2:**
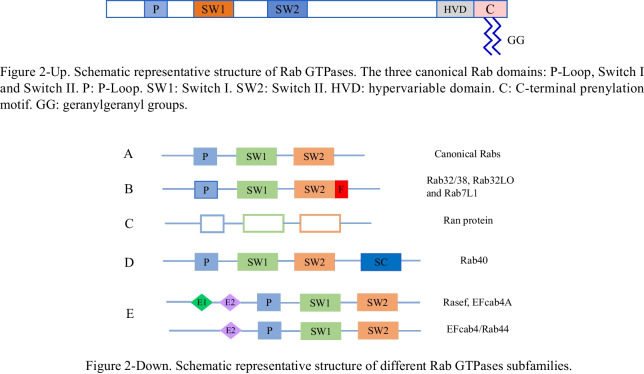
Schematic representative structure of different Rab GTPases subfamilies. **A** Most of Rabs contain three canonical Rab domains made of P-Loop, which is a nucleotide-binding motif fundamental for GTP/GDP cycling, Switch I and Switch II domains, acting as the interaction sites between other regulatory factors and Rab. **B** Rab32/38, Rab32LO and Rab7L1 subfamilies were characterized by an ultra-conserved FALK domain, downstream Switch I domain. **C** Ran proteins exhibited a distinctive protein sequence that is ultra-conserved in all the eukaryotes. **D** Rab40 proteins showed an additional SOCS box at the C-terminal region, which is considered fundamental for Varp proteasomal degradation in mammalian melanocytes. **E** Rab chimeras seem to be the result of the fusion of a canonical Rab at C-terminus with one or two calcium-binding EF-hand motifs at N-terminus. P: P-Loop. SW1: Switch I. SW2: Switch II. F: FALK motif. SC: SOCS. E1&E2: EF-hand motifs

Rab protein family members are highly conservative evolutionally with similar sequences, and are composed of multiple subfamilies that possess some common structural features, consisting of highly conserved G domains, amino and carboxyl ends with highly variable sequences and lengths. The G domain consists of five α-helix domains, six β-sheet domains and five cyclic peptides, and there are two switches in the G domain (I and II), acting as the interaction sites between other regulatory factors and Rab (Fig. 2). The carboxyl terminus of Rab protein contain different membrane localization structures, and among those structures, two cysteine are isopentenylated substrates, making Rab protein hydrophobic, which is necessary for reversible membrane adhesion of Rab proteins (Goody et al. 2005).

Rab proteins contribute to the promotion and regulation of anchoring and fusion of transport vesicles. The common characteristics of the Ras GTPases is to function as molecular switches of GTP. Indeed, Rabs in the GDP-bound form are mainly distributed in the cytoplasm, while Rabs in the GTP-bound form are located on the cell membrane, inner membrane and transport vesicle membrane, and regulate the formation of SNARE complexes.

The activity of Rab GTPases is regulated by GEFs and GAPs. The GEFs catalyzes the exchange of GDP with GTP, leading to the release of GDP and thereby the activation of Rabs (Hutagalung and Novick 2011). In contrast, the hydrolysis of GTP to GDP, converting Rabs from the GTP-bound state to the GDP-bound state, is not only driven by the intrinsic Rabs GTPase activity but is also catalyzed by GAPs (Stenmark 2009). Some GAPs participate in regulating specific Rab proteins, such as TBC1 domain family member 30 (TBC1D30), which only inactivates Rab8, whereas some GAPs function towards multiple Rab proteins. For example, TBC1 domain family member 4 (TBC1D4) inactivates Rab2A, Rab8, Rab10 and Rab14 (Fukuda 2011). GEFs can active Rabs, and are divided into several groups with specific domain characteristics, including Sec2p domain, DENN domain and Vps9p domain. Some GEFs are identified to be associated with Rab cascades, and are particularly recruited by one Rab protein to activate another Rab, which mediates the downstream pathway. One example is Ypt32/Rab11, which recruits Sec2/Rabin8 GEFs to the cell membrane, further activating Sec4/Rab8.

## Rabs and vesicle trafficking

Vesicles are the indispensable functional and structural components of the endomembrane system of cells with carrier functions and manifestations of the directional transport of intracellular substances. In eukaryotic cells, protein molecules are transported along the pathways of endocytosis and exocytosis. Vesicle transport starts from the budding of one organelle to another target organelle and unloads the transported substances through the process of recognition, docking and fusion.

The endocytic pathway associates the plasma membrane with the endosome and lysosome. In detail, endocytosis begins with the collection of cargo at the plasma membrane, followed by the formation and fission of an endocytic vesicle. Then these vesicles gradually fuse with early endosomes (EEs), a primary sorting station for membrane proteins and lipids.

In mammalian cells, several Rabs are localized to the endocytic pathway, and exert specific functions in different steps of endocytosis and recycling endosome pathways. As a marker for EEs, Rab5 is localized in EE and regulates the transport of endosome from plasma membrane to EEs (Bucci et al. 1992). Rab4, Rab11, Rab25 and Rab35 participate in recycling pathway. Particularly, Rab11 and Rab25 mediate recycling through the recycling endosomes (REs), while Rab4 and Rab35 facilitate fast recycling from EEs and REs to the plasma membrane directly (Bucci et al. 1992; Martinez-Arroyo et al. 2021). Afterwards, Rab7 regulates maturation of late endosomes (LEs) and conducts LE to the lysosome for degradation. However, Rab9 and Rab24 are involved in trafficking from LEs to the trans-Golgi network (TGN) (Bhuin and Roy 2014).

In the exocytic pathway, Rab1 and Rab2 regulate the trafficking between endoplasmic reticulum (ER) and the Golgi complex. Additionally, the action of Rab6 influences the intra-Golgi transport, while Rab8 and Rab11 control the trafficking from TGN to cell surface. Furthermore, the exocytic transport of secretory granules and vesicles from TGN to apical-lateral membranes is modulated by Rab3, Rab11, Rab27 and Rab38 (Bhuin and Roy 2014).

**Fig. 3:**
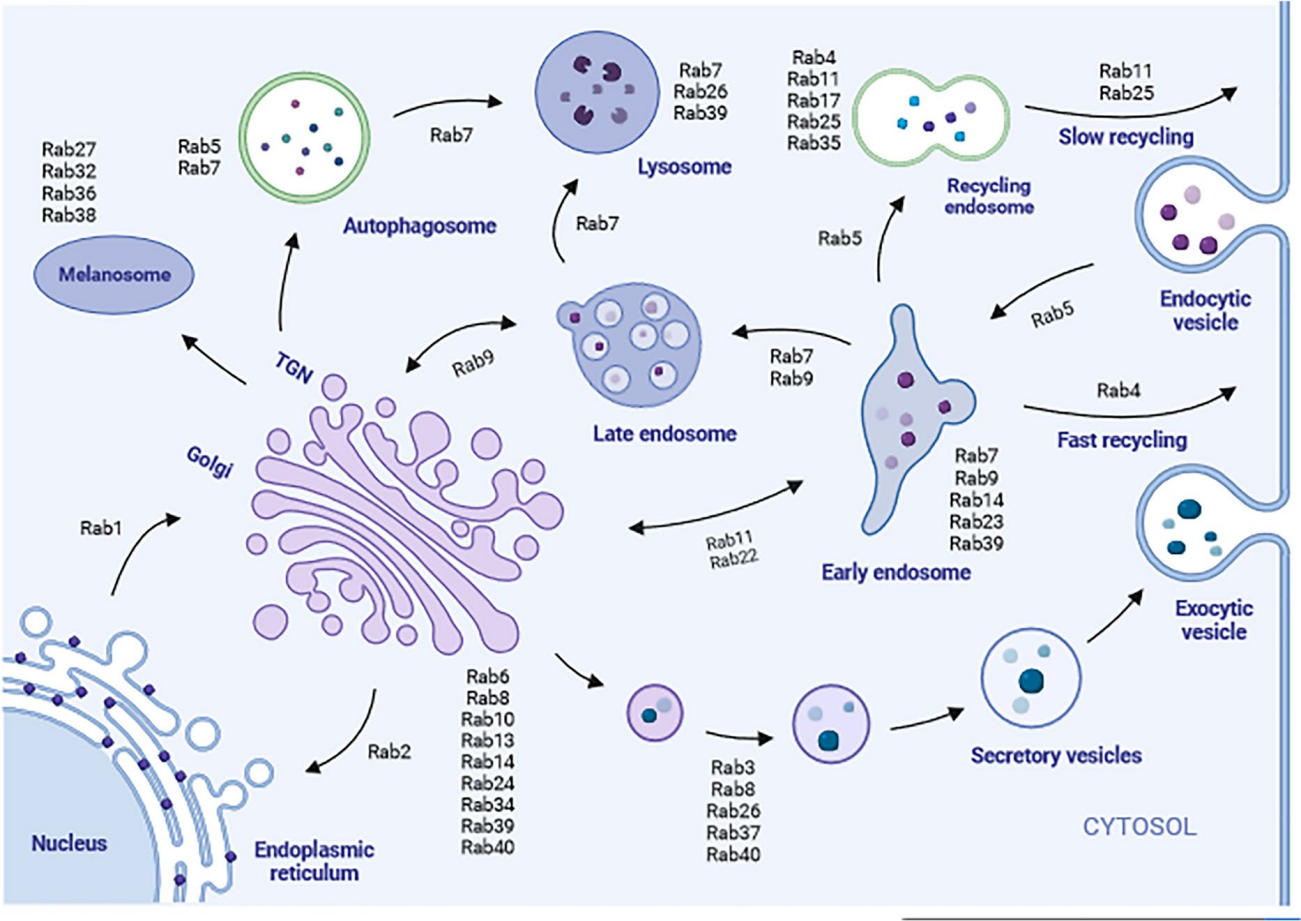
Subcellular localization of Rab GTPases and relative functions in vesicular transport. Highlighted Rab proteins that regulate the process of intracellular vesicular transport, including endocytosis, exocytosis and vesicles delivery between organelles. Rab1 mediates ER-Golgi traffic, while Rab2 is important in transport from ER to Golgi. Most early endocytic steps rely on Rab5, which mediates endosome fusion of vesicles to form the early endosome, and directs membrane transport from the early endosome to the recycling endosome. Rab7 and Rab9 are late endosomal GTPases, and regulate the maturation of LEs and their fusion with lysosomes, and the trafficking from LEs to TGN, respectively. Rab4 regulates fast endocytic recycling directly from EEs, whereas Rab11 and Rab25 are involved in slow endocytic recycling through REs. Rab11, together with Rab22, also regulate trafficking between EEs and the TGN. Several secretory vesicles use Rab3, Rab8 and Rab26, et al*.* to exocytose their cargo. The Golgi-localized Rab6, Rab33 and Rab40 regulate intra-Golgi transport of vesicles, while Rab8 mainly mediates the constitutive transport from the TGN to the plasma membrane. In addition, Rab27 is well-studied in the melanosome trafficking that also relies on Rab32 and Rab38. The vesicular movement regulated by Rab proteins is indicated by arrows. Created in biorender.com

In eukaryotic cells, Rabs interact with effector molecules at different stages in time and space, thereby playing a crucial role in the membrane transport system and controlling different transport pathways and organelle biogenesis, which is essential for the intracellular vesicle budding, vesicle motility and delivery, and anchoring and fusion at specific membranes via the recruitment of effectors (Fig. 3). Vesicular transport is an active transport process mediated by Rab proteins. In recent decades, prominent progresses have been made in studying the role of Rabs in membrane transport.

As is well known, the selection of cargo into differential trafficking vesicles is the basic mechanism in intracellular transport. Generally speaking, sorting is regulated by a combination of transmembrane proteins including cargo or cargo receptors, and cytosolic coat complexes (Stenmark 2009). Rab GTPases are important for recruiting specific coat to different intracellular membranes. For instance, the cytosolic tail of mannose-6-phosphate receptors (M6PRs) is recognized by TIP47, which is the sorting adaptor and an effector of Rab9. Rab9 recruits TIP47 to LE membranes and enhances the affinity of TIP47 for its cargo, thus promoting M6PRs sorting into LE recycling buds (Carroll et al. 2001). In addition, Rab5 has been recognized as an essential factor for cargo sequestration. Complexed with GDI, Rab5 participates in the formation of clathrin-coated pits and the clathrin-mediated endocytosis of transferrin receptors (McLauchlan et al. 1998).

## Rab GTPases in vesicle motility/delivery

The directionality and efficacy of vesicular transport are partially regulated by actin-dependent (myosins) and microtubule-dependent motors (kinesin or dynein). In recent decades, numerous researches have revealed the important roles of Rab GTPases and effectors in the mediation of this trafficking procedure. Actin motors of the myosin V family are identified to be correlated with cargo vesicles in a Rab-dependent manner. For instance, Rab27A recruits melanophilin, an adaptor protein, to melanosomes membranes. Myosin va27 thus links to the melanosomes, and these Rab27A-positive vesicles are then shuttles around the cell periphery, which is critical for the normal function of melanocytes (Ménasché et al. 2000). Functioning as an adaptor of myosin v, Rab11 family-interacting protein 2 (Rab11FIP2), the effector of Rab11, correlates Rab11A-positive endocytic recycling vesicles with myosin vb. In endocytic recycling pathways, myosin motors could be direct or indirect effectors of different Rab proteins, for example, myosin vb acts as a direct effector of both Rab 8A and Rab11A (Roland et al. 2007). Microtubule-dependent motors involved in membrane traffic, including plus-end-directed motors of the kinesin protein family and minusend-directed motors of cytoplasmic dynein, are modulated by Rab proteins. Kinesins act as either direct or indirect effectors according to different Rab GTPases (Stenmark 2009). For instance, KIF20A is an effector of Rab6 located in the Golgi apparatus while in the endocytic recycling KIF16B is recruited to the endosome membranes through combining with PtdIns-3-phosphate (PtdIns(3)P) (Hoepfner et al. 2005). In contrast, Dynein is an indirect effector for the Rab7 (Jordens et al. 2001).

## Rab GTPases in vesicle tethering and fusion

Another important function of Rab proteins is to assist the localization of transport vesicles for tethering and fusion with target membranes. The process of vesicle tethering to target membranes requires the involvement of tethering factors, consisting of two categories, long coiled helical chains including p115, Golgins and EEA, and multi-protein-complexes including TRAPPI and TRAPPII, also known as the effectors of Rab proteins (Li et al. 2021). The interactions of Rab proteins, tethering factors and multi-protein-complexes that have been discovered include Rab1/Ypt1 effector TRAPPI and TRAPPII complexes (Sacher et al. 2008), Rab5/Vps21 effector CORFET complex (Zhou et al. 2018) and SEC4/Rab8 effector exocatalyst complex (Yang et al. 2019a). The above tethering factors are typically recruited by Rab proteins and interact with SM proteins (Sec1-Munc18) to promote the assembly of SNAREs complexes for membrane fusion (Ohya et al. 2009). v-SNAREs, SNAREs on the vesicle membrane, and t-SNAREs, SNAREs at the target membrane form trans-SNARE complexes, thereby forming the fusion pores and promoting the final fusion of vesicles and target membranes (Bhuin and Roy 2014).

## Tumor initiation by Rab GTPases

Rab family members are crucial regulators of cellular membrane traffic, and in recent years, many Rabs have also been proposed as tumorigenic or metastatic biomarkers. Abnormal expression levels of Rab GTPases has been found in a variety of cancers including breast, liver and lung malignancies (Erol et al. 2024). The sub-cellular vesicle trafficking of cell adhesion receptor molecules (including integrins, cadherin-catenin) and proteases is required for cellular migration and invasion (Jin et al. 2021). Mutations of Rab genes and post-translational modifications of Rab proteins affect vesicle transport network by regulating tumorigenic potential, cell invasion and metastasis behavior (Gopal Krishnan et al. 2020). On the other hand, Rabs can also play tumor suppressive roles that induce apoptosis and inhibit angiogenesis (Gopal Krishnan et al. 2020).

Rab proteins and their effectors are demonstrated to be overexpressed or functionally deficient mutations in multiple diseases, including tumor progression (Qin et al. 2017).Cancer progression involves migration, invasion, autophagy, exosome secretion, metabolism and drug resistance (Jin et al. 2021). It has been observed that one same Rab acts in a context-dependent manner as either an onco-protein or a tumor suppressor in various tumors, which may be partially due to the interaction between one specific Rab with different effectors or cargos in different cancers (Tzeng and Wang 2016). A series of Rab GTPases such as Rab1, Rab3, Rab8, Rab25, Rab27 and others (as summarized in Table 2) promote cancer cells migration and invasion to exert critical infuences on tumor progression through mediating intracellular signaling pathways (Tzeng and Wang 2016).

**Table 2:** Summary of oncogenic Rab proteins in cancer research

Rab GTPases	Localization	Cancer type	Regulatory mechanisms	Refs
Rab1	Endoplasmic reticulum, Golgi	Liver	Rab1A promotes malignant growth and metastasis of HCC through enhancing hyperactive AA-mTORC1 signaling	Xu et al. 2015)
		Breast/Colon/Melanoma	PITPNC1 promotes vesicular secretion in malignancy by binding Golgi-resident PI4P and localizing RAB1B to the Golgi	Halberg et al. 2016)
Rab2A	Golgi, Autophagosome	Breast	Rab2A controls post-endocytic trafficking of MT1-MMP and regulates Golgi transport of E-cadherin	Kajiho et al. 2016)
Rab4A	Early endosome, Recycling endosome	Melanoma	By regulating procathepsin L secretion, Rab4A switches tumour progression induced by human melanoma cells in nude mice	Barbarin and Frade 2011)
Rab5	Early endosome, Early phygosome	Breast	EGFR vesicular recycling is regulated by Rab5 to promote migration in TNBC	Stallaert et al. 2018)
		Pancreatic	RAB5A promotes filopodia formation and migration in pancreatic cancer cells through the activation of cdc42 and β1-integrin	Yuan and Wei 2021)
		Cervical	Deficiency of Rab5a inhibits cell motility and invasiveness via integrin-mediated signaling pathway in cervical cancer	Liu et al. 2011)
Rab8	Plasma membrane,Golgi, Phagosome	Breast	Rab8 mediates the traffic of MT1-MMP to PM, and MT1-MMP-dependent collagen degradation and invasion in breast cancer cells	Bravo-Cordero et al. 2007)
Rab10	Endoplasmic reticulum, Endosomes	Liver	Rab10 deficiency inhibits the HGF pathway, while Rab10 overexpression is correlated with poor prognosis of HCC	Wang et al. 2017)
		Cervical	Rab10 is identified to facilitate the recycling of active integrin β1 to the cell surface	Jin et al. 2021)
Rab11	Recycling endosome, TGN	Gastric	Increased expression levels of Rab11 are closely associated with nodal metastasis in gastric cancer tissues	Dong et al. 2016)
		Cervical	Hypoxia promotes invasion and migration of cervical cancer cells via the Rab11 trafficking of integrin αvβ3/FAK/PI3K pathway	Xu et al. 2017)
		Breast	Hypoxia stimulates invasion of breast carcinoma cells through the Rab11 trafficking of the integrin alpha6beta4	Yoon et al. 2005)
Rab13	Recycling endosome, TGN	Colorectal	Rab13 can mediate its effects on the cell proliferation and invasiveness of colorectal cancer cells via autocrine and paracrine signaling	Hinger et al. 2020)
		Cervical	Rab13 facilitates integrin β1 recycling to the cell surface	Howe et al. 2020)
		Liver	Knockdown of RAB13 suppresses liver cancer cell proliferation and metastasis through inhibiting the PI3K/AKT signaling pathway, expression of CDK1/CDK4 and EMT process	Jiang et al. 2023)
Rab14	Endosomes, Golgi	Gastric	Rab14 is identified as oncogene and stimulates proliferation of gastric cancer cells through AKT signaling pathway	Guo et al. 2017)
		Ovarian	Rab14 promotes ovarian cancer cell proliferation through Wnt signaling pathway	Hou et al. 2016)
Rab18	Endoplasmic reticulum	Gastric	Rab18 stimulates gastric cancer growth and chemoresistance through regulation of mitochondrial function and survivin	Wu et al. 2018)
Rab21	Early endosomes	Breast /Cervical	Rab21 regulates the endosomal trafficking of integrin β1 and its overexpression increases cell migration and cancer cell adhesion	Pellinen et al. 2006)
Rab22	Early endosome, Recycling endosome	Liver	Rab22a is identified to promote the proliferation, migration and invasion of lung cancer through up-regulating PI3K/Akt/mTOR pathway	Wang et al. 2022)
		Breast	The formation of microvesicles that enhances breast cancer invasion and metastasis, is mediated by the HIF-dependent expression of RAB22A	Wang et al. 2014)
Rab25	TGN, Apical recycling endosomes	Breast	Rab25 mediates upregulation of anti-apoptotic pathways PI3K/AKT and downregulates pro-apoptotic molecules BAX and BAK1	Cheng et al. 2004)
		Non-small-cell lung cancer	The integrin β1 recycling medicated by Rab25 activates AKT/β-catenin pathway, thus promoting erlotinib resistance in non-small-cell lung cancer	Ménasché et al. 2000)
		Ovarian	Rab25-medicated integrin β1 promotes cell invasion through EGFR/VEGF/Snail axis	Roland et al. 2007)
Rab27	Endosomal exocytic vesicles	Breast	Rab27b is identified to activate MMP2 secretion and enhance breast cancer cell invasion in the ERC breast cancer	Hendrix et al. 2010)
		Breast	Rab27a blockade decreases MMP9 secretion and primary tumor growth, and lung dissemination of a metastatic carcinoma	Bobrie et al. 2012)
		Pancreatic	ZIP4 promotes growth of orthotopic pancreatic tumors in mice and loss of muscle mass by activating CREB-regulated expression of RAB27B,required for release of extracellular vesicles	Yang et al. 2019b)
Rab31	Late endosome, Trans Golgi, TGN	Cervical	Rab31 stimulates the invasion and metastasis of cervical cancer cells through enhancing EMT and inhibiting MAPK6 degradation	Huang et al. 2022)
		Colon	In colon cancer, RAB31 promotes tumor progression by regulating the secretion of HGF in the tumor stroma	Yang et al. 2020)
Rab32	Late endosome, Lysosome	Liver	Rab32 associates with lysosomes and regulates proliferation and metabolism through supporting mTORC1 signaling	Drizyte-Miller et al. 2020)
Rab35	Plasma membrane, Endosomes	Breast /Cervical	Rab35 enhances the expression of integrin β1 and promotes cell migration in cervical and breast cancer	Argenzio et al. 2014)
Rab40B	TGN-derived vesicle	Breast	Rab40b regulates the transport of MMP2/9 during invadopodia formation and metastasis in breast cancer	Jacob et al. 2013)

### Breast cancer

As a common kind of malignancy globally, breast cancer ranks the second most prevalent leading cause of tumor-related mortality in women (Xu and Tang 2021). High Rab2A expression is found in human breast cancer suggesting Rab2A could be an independent predictor of disease recurrence in breast cancer patients (Kajiho et al. 2016). More importantly, by binding with VPS39, Rab2A regulates post-endocytic trafficking of most notably membrane type 1 (MT1)-MMP, an important metalloprotease for matrix remodeling and invasion. Besides, it is further associated with Golgi transport of E-cadherin (Kajiho et al. 2016).

In breast cancer cell MDA-MB-231, the exocytosis of MT1-MMP occurs during migration into three-dimensional collagen matrices (Zhang et al. 2020). Mechanistically, Rab8-activated mutant induces MT1-MMP exocytic transport, collagen degradation and invasion, whereas downregulation of Rab8 expression inhibits these processes (Dong et al. 2016). Therefore, the delivery to invasive structures and pro-invasive activity of MT1-MMP are mediated by Rab8 GTPase.

Tumor cells can survive in stressful microenvironments autonomously, such as hypoxia (Chan et al. 2012). Hypoxia has been identified to increase breast cancer cell invasion through the modulation of Rab11 (Colombo et al. 2006). Dominant-negative mutant of Rab11 dramatically inhibits hypoxia-induced invasion without affecting cell apoptosis in breast cancer. Furthermore, hypoxia induces a significant promotion in α6β4 surface expression, which is relied on Rab11 and stable microtubules.

Pellinen et al*.* have demonstrated that the endo/exocytic traffic of integrins are regulated by the interaction between Rab21 and the cytoplasmic domains of α-integrin chains (Pellinen et al. 2006). Besides, this process is relied on Rab21 GTP/GDP cycle and correct membrane targeting. Knockdown of Rab21 inhibits integrin-mediated breast cancer cell adhesion and motility, and conversely, overexpression of Rab21 promotes cell migration and adhesion (Pellinen et al. 2006).

In breast cancer patients, overexpression of Rab22A in the primary tumor is correlated with reduced overall and metastasis-free survival. Exposure of breast cancer cells to hypoxia enhances microvesicle (MV) shedding, which is regulated by HIF-dependent expression of Rab22A (Wang et al. 2014). Besides, *Rab22A* deficiency *inhibits* metastasis in an orthotopic mouse model of breast cancer. Hendrix et al*.* have found that overexpression of Rab27B stimulates cell cycle transition from G_1_ to S phase, cell growth and invasiveness in ER-positive breast cancer cells (Bravo-Cordero et al. 2007). In addition, secretion of Heat-shock protein 90α (HSP90α) is relied on Rab27B and is required for cell proliferation and invasion (Bravo-Cordero et al. 2007).

In breast cancer cells, high expression of Rab31 stimulates cell proliferation and promotes cells to switch from an invasive phenotype to a proliferative phenotype by elevating cell growth, lessening adhesion and decreasing invasion (Grismayer et al. 2012). Furthermore, Rab31 overexpression impairs capacity to form lung metastases in vivo (Grismayer et al. 2012).

### Cervical cancer

Cervical cancer is one of the most prevalent tumors in women. In 2020, there are approximately 604,000 new cases and 342,000 deaths of cervical cancer worldwide (Huang et al. 2022). A previous study has shown that Rab5a expression is increased in cervical cancer tissues, and Rab5a knockdown markedly inhibits cancer cell proliferation and invasion (Liu et al. 2011). Mechanistically, the absence of Rab5a expression downregulates the assembly and activities of integrins and the downstream signaling molecules in cervical cancer cells, including phosphorylation of FAK and paxillin (Liu et al. 2011).

Rab11 and activation of Rac1 could promote cervical cancer cell migration and invasion in hypoxia (Xu et al. 2017). Moreover, hypoxia significantly increases the surface expression of αvβ3 integrin, which is dependent on Rab11. Rab31 is overexpressed in cervical cancer tissues and enhances migration and invasion of cervical cancer cells by promoting epithelial mesenchymal transition (EMT) and affects the cytoskeletal rearrangement in an MAPK6-dependent manner (Huang et al. 2022). In addition, knockdown of Rab31 impairs tumor growth and metastasis through MAPK6 in a xenograft mouse model (Huang et al. 2022).

### Colorectal cancer

Colorectal cancer (CRC), as the third most prevalent malignancy worldwide, comprises of a group of histologically heterogeneous diseases with different tumorigenic pathways. Over the past decade, with the advancement of sequencing techniques, great efforts have been made to reveal the molecular complexity behind initiation and progression of CRC.

High Rab3C expression levels promote tumor metastasis, which is associated with poor prognosis in CRC by modulating the secretion of IL-6 through exocytosis and activating the JAK2-STAT3 signaling pathway (Chang et al. 2017). Furthermore, Ruxolitinib, a JAK2-specific inhibitor, is demonstrated to decrease the migration ability and phosphorylation of STAT3 in Rab3C-overexpressing CRC cells (Chang et al. 2017). In CRC cells, StRIP3, a stitching peptide, selectively combines with Rab8A in its activated GppNHp binding state to suppress Rab8A-effector interactions, demonstrating an affinity comparable to that of Rab8A effector OCRL1 (Spiegel et al. 2014). This strategy may reveal a novel path to guide the targeting of Rabs for regulating effector binding.

Rab11 has been shown to interact with E-cadherin, and these molecules are indicators of poor survival time in CRC. Overexpression of Rab11 promotes cell migration by the elevated E-cadherin distribution, thereby enhancing connections between cells (Chung et al. 2016). Additionally, Rac1 activity is up-regulated and MMP2 expression is elevated in Rab11-overexpressing colon cancer cells (Chung et al. 2016). Rab13 has been confirmed to regulate small extracellular vesicles (sEVs) secretion in a KRAS-dependent mechanism in CRC, thus promoting proliferation and tumorigenesis, while deficiency of *Rab13* blocks these effects (Hinger et al. 2020). Overexpression of Rab31 in cancer-associated fibroblasts (CAFs) enhances cell migration in an HGF/MET-dependent manner in colon cancer cells, indicating a key role for Rab31-expressed CAF in the colon cancer cell migration by mediating paracrine secretion of HGF (Yang et al. 2020).

### Gastric cancer

As one of the most prevalent tumors in the world, gastric cancer (GC) occurs with a low 5-year survival rate of < 24% due to the trend of early invasion and metastasis (Guo et al. 2017). GC is a multifactorial disease, both environmental and genetic factors function in its etiology (Xu and Tang 2021).

The AKT kinase signaling pathway has been reported to be constitutively active in GC and stimulates cellular survival and tumorigenesis (Kang et al. 2042; Shi et al. 2014). A previous study has shown that Rab14 remarkably induces cell proliferation in GC cells. Moreover, deficiency of *Rab14* results in a significant cell cycle arrest from G1 to S transition and an apparent increase rate of early and late apoptosis in GC cells. Mechanistically, knockdown of Rab14 decreases the phosphorylation of AKT at serine 473, whereas total AKT expression remains unchanged, engendering a lower level of Cyclin D1 and CDK2 with reduced phosphorylation levels of Cyclin D1 at Thr286 (Guo et al. 2017).

The expression of Rab18 protein is upregulated in GC tissues and is associated with advanced stage and poor prognosis of GC (Wu et al. 2018). Besides, overexpression of Rab18 enhances GC cell growth, upregulates S phase percentage and positively regulates cellular migration and invasion. Mechanistically, after cisplatin treatment, Rab18 maintains cell viability and reduces cell apoptosis, with reduced mitochondrial reactive oxygen species (ROS) levels and elevated mitochondrial membrane potential (Wu et al. 2018).

Nambara et al*.* have reported that Rab27b is overexpressed in GC cells, and knockdown of Rab27b decreases the secretion of exosomes (Nambara et al. 2023). Additionally, *Rab27b* deficiency reduces peritoneal metastasis in a xenograft mouse model without affecting the proliferation or invasion ability of cancer cells (Nambara et al. 2023). Similarly, Rab31 is identified to be upregulated in GC tissues, which predicts poor survival in patients (Tang et al. 2018). Moreover, loss of *Rab31* suppresses tumor growth in a nude mouse model, while *Rab31* deficiency through glioma-associated oncogene homolog 1 (GLI1) suppresses cell motility, promotes apoptosis, and regulates the expression levels of cell cycle and apoptotic proteins in vitro (Tang et al. 2018).

### Liver cancer

Hepatocellular carcinoma (HCC) is one of the most prevalent and lethal tumors all over the world, with a high recurrence rate according to current treatment methods.

In HCC cells, *Rab1A* is identified as a direct target of miR-15b-5p by using bioinformatics and luciferase reporter assays, and deficiency of *Rab1A* also inhibits cell migration, and promotes apoptosis and endoplasmic reticulum stress (ERS) (Yang et al. 2015). Likewise, knockdown of Rab10 is found to suppress cell proliferation and colony formation but enhance cell cycle arrest and apoptosis in HCC cells, and is also identified to inhibit HCC growth in nude mice. Mechanically, PathScan results showed the decreased phosphorylation levels of the RTK family members (including InsR, Met/HGFR, Ron/MST1R, Ret) and elevated phosphorylation levels of stress and apoptosis family members (including HSP27, p38 MAPK and TAK1), indicating that inhibition of RTK pathways and activation of stress and apoptosis might be responsible for Rab10 modulation of HCC cell growth and apoptosis (Wang et al. 2017). Furthermore, Rab10 upregulation is correlated with a poor prognosis in HCC patients (Wang et al. 2017). Like Rab10, Rab11a overexpression enhances cell growth, migration, invasion, and anti-apoptosis in HCC cells. Besides, results from nude mice xenograft indicate that Rab11a can promote HCC cell growth in vivo. Further studies have suggested that Rab11a upregulates the expression of MMP2 by activating PI3K/AKT pathway, and LY294002, the PI3K/AKT pathway inhibitor, can suppress MMP2 expression (Zhang et al. 2020). Moreover, a recent study suggests that knockdown of Rab13 inhibits HCC cell growth and metastasis through inhibiting the PI3K/AKT pathway, CDK1/CDK4 expression and EMT (Jacob et al. 2013). Moreover, in *Rab13*-silenced HCC, sorafenib, a ferroptosis inducer, promotes GPX4-dependent ferroptosis. RAB13 is identified to be associated with ferroptosis vulnerability and metabolism-relevant signaling. And knockdown of RAB13 increases sensitivity to sorafenib, which is correlated with intracellular iron accumulation and elevated lipid oxidation levels. Additionally, alterations in ferroptosis vulnerability induced by RAB13 are found to be dependent on GPX4 expression (Jacob et al. 2013).A previous study has shown that, consistent with the attenuation of mTORC1 activity, knockdown of Rab32 suppresses cell proliferation while increases the nuclear localization of TFEB and lysosomal biogenesis in HCC cells (Drizyte-Miller et al. 2020). Besides, *Rab32* deficiency results in a reduction in the lysosome correlation with mTOR, regulatory-associated protein of mTOR and mTORC1 pathway proteins, including RagC and Lamtor1 (Drizyte-Miller et al. 2020).

### Lung cancer

Lung cancer is recognized to be the most lethal malignant tumors all over the world, with an 5%-increase of incidence rate every year. Upregulation of Rab1a expression in lung cancer cells enhances cell mobility including migration, invasion and metastasis both in vitro and in vivo through activating JAK1/STAT6 signaling pathway in an IL-4Rα dependent manner (Huang et al. 2021). In addition, Rab1A is found to be a determinant sensitivity of JAK1 inhibitor, suggesting that JAK1 inhibitor could be potential therapeutics for lung cancer metastasis (Huang et al. 2021). Hypoxia promotes Rab5 activity through HIF1α, and simultaneously gives rise to the re-localization of Rab5 to focal adhesion proteins (FAs) in lung cancer cells. Moreover, hypoxia is clarified to elevate FAK phosphorylation status and subsequently the Rac1 activity, and migration of lung cancer cells is dependent on Rab5 activity (Silva et al. 2016). Overexpression of Rab11a is correlated with positive nodal status, advanced Tumor Node Metastasis (TNM) stage and poor patient prognosis. Additionally, Rab11a is identified to promote lung cancer cell growth, migration and invasion through modulating Hippo signaling pathway (Dong et al. 2017). Rab22a is identified to interact with PI3K85α, an important factor controlling flux via PI3K signaling. Moreover, Rapamycin, the mTOR inhibitor, is proved to effectively suppress Rab22a-triggered proliferation, migration and invasion in lung adenocarcinoma cells, indicating that Rab22a regulates the biological malignancies of lung adenocarcinoma cells through activating PI3K/AKT/mTOR signaling (Wang et al. 2022).

### Ovarian cancer

Derived from human epithelial and germ cells, ovarian cancer is one kind of malignant tumors, ranking the second most common gynecological malignancywith the highest mortality rate (Colombo et al. 2006).

Compared with the ES‑2 ovarian cancer cell line that is cisplatin‑sensitive, overexpression of Rab25 promotes PI3K/AKT signaling in SKOV‑3 ovarian cancer cells that are cisplatin‑resistant (Fan et al. 2015). In contrast, *Rab25* deficiency or administration with LY294002, significantly elevates the sensitivity of these ovarian cancer cells to cisplatin (Fan et al. 2015). Hou et al*.* have reported that *Rab14* deficiency inhibits cell proliferation and invasion (Grismayer et al. 2012). Moreover, overexpression of Rab14 regulates GSK3β phosphorylation and β-catenin accumulation in nucleus. Knockdown of Rab14 suppresses TCF transcriptional activity with corresponding changes in target genes of Wnt signaling pathway, including *MMP7* and *c-Myc* (Hou et al. 2016). Thus, Rab14 stimulates ovarian cancer proliferation, invasion and chemoresistance by activating GSK3β/Wnt signaling.

Additionally, knockdown of Rab22a inhibits invasion and migration of epithelial ovarian cancer cells, increases E-cadherin expression but suppresses N-cadherin expression (Zhang et al. 2014). Moreover, Rab22a overexpression abrogates the miR-373 effects on suppressing cell invasion and migration in epithelial ovarian cancer cells (Zhang et al. 2014).

## Tumor suppression by Rab GTPases

Contrary to the role of Rab proteins in positively regulating carcinogenesis, a minor portion of Rab proteins is observed to function as a suppressor that exerts negative effects on cancer cells through inducing cancer cell apoptosis but inhibiting angiogenesis (Gopal Krishnan et al. 2020). The Vascular Endothelial Growth Factor Receptor 1 (VEGFR1) and VEGFR-2 derived from REs and secreted towards the cell membrane is depending on Rab11 vesicles, suggesting that Rab11 plays a crucial role in neo-angiogenesis (Roma-Rodrigues et al. 2023). Rab11 is also found to participate in the progression of angiogenic sprouts formation through linking to the phosphorylated vascular endothelial cadherin (p-VE-Cadherin), as well as in the recycling of 5-integrin-p-FAK complexes, which is associated with the assembly of adhesion sites in endothelial cells (Benwell et al. 2021). Rab35 is involved in regulating actin assembly in the period of sprouting angiogenesis, especially controlling actin dynamics during angiogenesis (Francis et al. 2022). In detail, GEF DENNd1c connects activated Rab35 to the actin cytoskeleton. Upon moving to actin, Rab35 restricts actin polymerization and further remodeling that is essential for sprout formation (Francis et al. 2022). Deconstruction of the signaling mechanisms regulated by Rab GTPases during apoptosis and angiogenesis may provide potential therapeutic targets for cancer cells.

The precisely context-dependent dual character of Rabs functioning as either a tumor initiator or a tumor suppressor might be partially due to the binding with specific effectors in multiple tumors (Tsai et al. 2014). Some studies demonstrate that Rab proteins including Rab1, Rab11, Rab23 and Rab25, may play tumor suppressive roles in several kinds of tumors (as reviewed in Table 3).

**Table 3:** Summary of Rab proteins functioning as tumor suppressor in cancer research

Rab GTPases	Localization	Cancer type	Regulatory mechanisms	Refs
Rab1	Endoplasmic reticulum, Golgi	Breast	RAB1B functions as a metastasis suppressor in Triple-negative breast cancer via regulating the TGF-β/SMAD signaling pathway	Jiang et al. 2015)
Rab7	Autophagosome, Late endosomes	Prostate	Rab7 facilitates troglitazone to prevent HGF-induced protease secretion, and prostate tumor growth and invasion	Steffan et al. 2014)
Rab17	Recycling endosome, Melanosome	Liver	Rab17 inhibits the proliferation and migration of hepatocellular carcinoma cells and reduces the tumor growth through ERK signaling pathway	Wang et al. 2015)
Rab23	Autophagosome, Plasma membrane	Breast	Rab23 can inhibit cell growth and proliferation and induces cell apoptosis, due to the inhibition by Rab23 of Gli1 and Gli2 expression in breast cancer cells	Liu et al. 2015)
Rab25	TGN, Apical recycling endosomes	Skin squamous cell	Rab25 deficiency promotes the development and neoplastic transition of skin squamous cell cancer by the dysregulation of integrin trafficking	Jeong et al. 2019)
		Colon cancer	In colon cancer, the loss of Rab25 significantly reduces β1 and promotes its distance from the lateral membranes	Nam et al. 2010)
		Esophageal squamous cell carcinoma	Overexpression of Rab25 exhibits anti-invasive and anti-tumorigenic properties through down-regulation of the FAK-Raf-MEK1/2-ERK signaling pathway in ESCC	Tong et al. 2012)
Rab26	Autophagosome, Lysosome	Breast	Rab26 modulates the autophagic degradation of phosphorylated Src by bindting with ATG16L1, thus suppressing the migration and invasion of breast cancer cells	Liu et al. 2021)
Rab27	Endosomal exocytic vesicles	Non-small-cell lung cancer	Overexpression of Rab27a increases exosome secretion and induces efficient antitumor immune effects, thus suppressing tumor formation in a tumor mouse model	Li et al. 2013)
Rab37	Secretory granule	Lung	RAB37 is a metastasis suppressor via regulating TIMP1 for exocytosis to inhibit MMP9 invasion signalling	Tsai et al. 2014)
		Nasopharyngeal	RAB37 regulates the exocytosis of TIMP2 to inactivate MMP2, thereby suppressing tumor invasion in nasopharyngeal cancer	Li et al. 2018)
Rab45	Perinuclear region	Leukemia	Overexpression of Rab45 cDNA induces apoptosis through the loss of MMP in CML cells	Nakamura et al. 2011)

Rab1B is observed to be aberrantly down-regulated in triple-negative breast cancer (TNBC), and *Rab1B* deficiency stimulates the proliferation and migration of TNBC cells by diminishing degradation of ubiquitin, elevating phosphorylation levels of SMAD3 and up-regulating TGF-β-triggered EMT (Jiang et al. 2015), which increases the expression of TGF-β receptor 1 (TβR1). Tumour suppressor phosphatase and tensin homologous 10 (PTEN) has been found to dephosphorylate Rab7 on two conserved amino acid residues including serine 72 and tyrosine 183, which are crucial for GDP dissociation inhibitor (GDI)-mediated recruitment of Rab7 to late endosomes as well as subsequent endosomes maturation (Shinde and Maddika 2016). Therefore, PTEN-induced endosome maturation through regulating Rab7 phosphorylation levels is considered as an efficient method of controlling EGFR signaling. In mature epithelia, tumorigenic signaling is limited to appropriate homeostatic levels by Rab11 endosomes to maintain normal tissue growth and turnover. Decreased expression levels of Rab11 are associated with advanced-stage tumor as well as poorer survival rates. In addition, *Rab11* deficiency leads to a rapid expansion of intestinal stem cell pool, with cells autonomously activating Yki/Yap. Mechanistically, *Rab11*-deficient intestinal tumors exhibit a significant increase in upd3/IL6-Stat3, nuclear Yap and amphiregulin-MAPK signaling (D'Agostino et al. 2019).

A previous report has shown that Rab23 directly correlates with Su(Fu) and suppress Gli1 activity through Su(Fu) (Chi et al. 2012). In breast cancer cells, activated Rab23 suppresses cell growth and represses DNA synthesis but induces cell apoptosis. Furthermore, the above effects have been proven to be due to the Rab23-mediacted expression inhibition of Gli1 and Gli2, suggesting Rab23 might be a potential therapeutic target for breast cancer (Liu et al. 2015). Tong et al. have found that in esophageal squamous cell carcinoma (ESCC), low expression levels of Rab25 are linked to decreased overall survival (Tong et al. 2012). *Rab25* deficiency in both ESCC cells and in vivo clinical samples is correlated with hypermethylation of *Rab25* gene promoter region. Furthermore, Rab25 exerts anti-invasion and anti-angiogenesis functions by deregulating FAK-Raf-MEK1/2-ERK signaling pathway (Tong et al. 2012). Overexpression of Rab27a increases exosome secretion and elicits efficient antitumor immunity, thus suppressing tumor formation in a tumor mouse model. This finding would provide new insights into the development of tumor vaccines based on efficient exosome (Li et al. 2013).

Rab37 mediates the exocytosis of tissue inhibitor of metalloproteinase 1 (TIMP1) in a nucleotide-dependent way to disrupt MMP9 migration in lung cancer (Tsai et al. 2014). By contrast, dysfunction of Rab37 or TIMP1 abolishes metastatic suppression of lung cancer cells. Patients suffering from lung cancer with metastasis and poor survival demonstrates that decreased expression levels of Rab37 are consistent with low TIMP1 expression in lung tumors (Tsai et al. 2014). Therapeutic strategies such as alterations in DNA demethylation status of *Rab37* gene and elevated stability of RAB37 protein may contribute to the development of cancer therapy (Sundberg et al. 2011). Moreover, phosphorylation on threonine 172 (T172) negatively regulates Rab37 activity, and impairs the Rab37-induced exocytosis of TIMP1, resulting in the suppression activity of Rab37 on lung cancer cell motility (Tzeng et al. 2017).

A previous publication has reported that Rab39a is frequently downregulated in poorly differentiated or with lymphatic node metastasis compared with matched non-cancerous tissues, and is linked to high metastasis rate of lymphatic node and poor survival of patients (Zou et al. 2019). Moreover, Rab39a hardly affects cell viability but markedly inhibits cell mobility including migratory and invasive capacities as well as EMT process in cervical cancer. Mechanistically, Rab39a acts as a potential tumor suppressor via significantly decreasing phosphorylation levels of AKT at Ser473, and its inhibition effects could be blocked by using AKT pathway inhibitor (Zou et al. 2019).

## Conclusions and perspectives

**Fig. 4:**
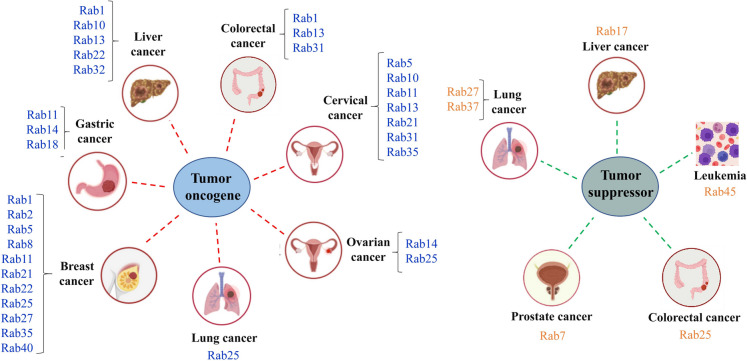
Schematic illustration of oncogenic and tumor suppressor Rab proteins in different cancers

In summary, Rab GTPases play an important role in temporal and spatial regulation of cellular membrane transport including several processes such as endocytosis, exocytosis and exosome secretion as well as vesicles delivery between organelles. Mechanistically, the functions of Rab proteins are coordinately regulated through cascades, involving shared effectors and regulatory proteins, and respond to cellular demand. Accumulating evidence has demonstrated that a large fraction of Rabs and Rab-associated factors influence tumorigenesis and metastasis through regulating intracellular signal transduction (Fig. 4). Thus, targeting particular Rab GTPases to adjust membrane trafficking might provide novel therapeutic approaches to cancer treatment.

## Data Availability

All data generated or analyzed during this study are included in this published article.

## Abbreviations

*AA*: Amino acid
*CDK1*: Cyclin-dependent kinase 1
*CML*: Chronic myelogenous leukemia
*EGF*: Epidermal growth factor
*EGFR*: Endothelial Growth Factor Receptor
*ERK*: Extracellular signal-regulated kinase
*FAK*: Focal adhesion kinase
*HGF*: Hepatocyte growth factor
*GPX4*: Glutathione peroxidase 4
*HIF*: Hypoxia-inducible factor
*KRAS*: Kirsten rat sarcoma viral oncogene
*MAPK*: Mitogen-activated protein kinase
*MEK*: Mitogen activated protein kinase 1/2, ERK 1/2
*MET*: Mesenchymal to epithelial transition factor
*MMP2*: Matrix metalloproteinase-2
*MMP9*: Matrix metalloproteinase-9
*MT1-MMP*: Most notably membrane type 1-matrix metalloproteinase
*mTORC1*: Mammalian/mechanistic target of rapamycin complex 1
*OCRL1*: Lowe syndrome protein
*PI3K*: Phosphatidylinositol-3-kinase
*Raf*: Rapidly accelerated fibrosarcoma
*RagC*: Ragulator complex protein
*TFEB*: Transcription factor EB
*upd3*: Unpaired 3
